# Exploring endometriosis through 3D *in vitro* models: A narrative review

**DOI:** 10.1016/j.mtbio.2026.103032

**Published:** 2026-03-17

**Authors:** Cara Juli, Edward Mairura Nyang'au, Martin Götte, Frauke von Versen-Höynck

**Affiliations:** a Department of Obstetrics, Gynecology and Reproductive Medicine, Division of Gynecologic Endocrinology and Reproductive Medicine, Hannover Medical School, Hannover, 30625, Germany; b Gynecology Research Unit, Hannover Medical School, Hannover, 30625, Germany; c Department of Gynecology and Obstetrics, Münster University Hospital, Münster, 48149, Germany; d Cells-in-Motion Interfaculty Centre (CiMIC,) University of Münster, Münster, Germany

**Keywords:** Endometriosis, 3D *in vitro*, Spheroids, Organoids, Bioprinting, Organ-on-a-chip, Explants

## Abstract

Endometriosis is a prevalent inflammatory disease affecting approximately one in ten women, characterized by endometrial tissue outside the uterus. Despite its high incidence, treatment options are limited, and the underlying pathology remains poorly understood. Reliable models are essential for investigating the mechanisms of endometriosis, necessitating an updated review of 3D *in vitro* models that better mimic the disease environment.

This review aims to provide a comprehensive overview of existing 3D *in vitro* models of endometriosis, focusing on their evolution, refinement, and application in understanding disease mechanisms and therapeutic screening.

A systematic search was conducted in the PubMed database for original and review articles published in English until July 2025. Search terms included "endometriosis," "3D model," "spheroid," "organoid," "microfluidic system," "organ-on-a-chip," "bioprinting," "chorioallantoic membrane," "amniotic membrane culture," and "explant." Exclusion criteria included non-English articles and studies not focused on human or relevant animal models.

Six major categories of 3D *in vitro* endometriosis models were identified, each with distinct structural and functional features tailored to specific research questions. Their evolution over time is discussed, along with critical evaluation of their limitations and practical challenges.

The insights gained from 3D *in vitro* models have significant implications for clinical practice, potentially informing targeted therapies and improving patient outcomes. For reproductive health specialists and scientists, these models may lead to more effective diagnostic and treatment strategies, thereby advancing the field. We discuss the future perspectives, of advancing and integrating these models into personalized medicine approaches and exploring novel therapeutic targets.

## Highlights

• Emerging bioengineering technologies coupled with AI enhance T3D *in vitro* systems to replicatenative endometrial architecture.
• 3D *in vitro* models harbour potential for personalized medicine
• 3D *in vitro* models are a critical advancement toward more effective endometriosis therapies

## Graphical abstract

Endometriosis is modeled in 3D in-vitro systems, to gain insights into the pathophysiological aspects of the disease, the prerequisite for the development of therapeutic strategies. Created in BioRender.com.

**Figure:**
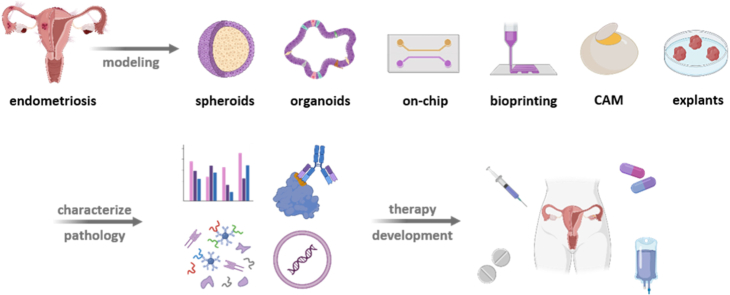

## 1 Introduction

### 1.1 Background on endometriosis

Endometriosis is a complex, chronic and inflammatory gynecological disease, marked by the presence of endometrial-like tissue outside the uterus, mainly occurring at the peritoneum, ovaries and other pelvic structures. Approximately 10 % of women in their reproductive years are affected, which are about 190 million women worldwide [1]. The disease is not only accompanied by several severe symptoms such as chronic pelvic pain, dysmenorrhea and dyspareunia, but is also associated with infertility and poorer pregnancy outcomes [2]. In connection with endometriosis and also other gynecological diseases, the term “women” is often used, however it is important to recognize that all individuals with a uterus can be affected. In rare cases, endometriosis in biological men undergoing steroidal therapy for prostate cancer have been described [3]. In many cases, endometriosis significantly impacts the quality of life, since many symptoms during and between menstruations hinder participation in daily activities. Due to the unspecific symptoms and the fact that the complaints of many women are not taken seriously, on average seven years pass between the first symptoms and a diagnosis [1]. Despite the high prevalence, the exact mechanisms of pathogenesis remain unclear. There are several formation theories, none of which have been proven, but retrograde menstruation is the most widely accepted one. Accordingly, viable endometrial tissue moves during the menstruation via the fallopian tubes into the pelvic cavity and attaches to peritoneal mesothelial cells. This allows the tissue to proliferate, invade pelvic structures, and form lesions [4]. Endometriotic cells may also be distributed to ectopic sites via lymphovascular metastasis [5]. However, the retrograde menstruation theory alone does not fully account for the occurrence of rare forms of the disease [6]. While the embryonic rest theory and the concept of coelomic metaplasia characterize routes of pathogenesis linked to aberrant cell differentiation [7,8], the tissue injury and repair hypothesis and the denervation-reinnervation theory are based on tissue damage and subsequent endometrial tissue release linked to mechanical activity such as hyperperistalsis of the uterus [9,10]. More recent concepts include the hypothesis of an involvement of endometrial stem cells in the disease, which could promote endometriosis due to their unlimited proliferative potential and developmental plasticity [11,12]. In addition, systemic inflammation, estrogen dependence and progesterone resistance, genetic and epigenetic alterations, aberrant angiogenesis and metabolic disorders are among the key factors involved in the development of endometriosis [11,13]. To gain a deeper understanding of the complex underlying pathogenetic mechanisms, models that accurately reflect the disease's main characteristics and enable the study of its formation and progression, are urgently needed. This knowledge is essential for advancing new diagnostics and therapeutic approaches. In line with this goal, this review highlights the key milestones in the development of three-dimensional (3D) *in vitro* models, providing an overview of the current state of research and unresolved questions in endometriosis studies.

### 1.2 Importance of 3D *in vitro* models

Endometriosis research tools should incorporate key disease features, allow to track cellular as well as molecular interactions and reflect the clinical patterns found in patients. Even if *in vitro* models do not capture all biological aspects of endometriosis, they allow to analyze essential cellular and molecular mechanisms, test potential treatments and provide a more accessible, cost-effective and ethically favorable research approach. Hereby, 3D endometriosis models offer several advantages over traditional two-dimensional (2D) cell cultures by more accurately replicating the complex microenvironment of endometriotic lesions. While 2D models grown as a monolayer and lack tissue like-architecture, 3D models better preserve cellular interactions, spatial organization and extracellular matrix composition, leading to more physiologically relevant results [14]. Furthermore, 3D endometriosis models offer multiple benefits compared to animal models. *In vivo* animal studies may provide insights into systemic interactions, however, the reproductive physiology of several experimental animal models, including rodents, differs substantially from humans [15]. 3D models offer a more ethical and cost-effective alternative with a higher human relevance for studying endometriosis pathophysiology and treatment approaches. Additionally, 3D *in vitro* models allow the culture of patient-derived material in a controlled microenvironment, enabling personalized medicine and more accurate disease stratification [16]. In this review, we describe the most important 3D *in vitro* models for endometriosis available to date as well as the results and insights that have been gained from these models so far. Table 1 provides an overview of the models, which are discussed in detail in the next section.

**Table 1:** Summary of the highlighted 3D *in vitro* endometriosis models, including the used material, methods and key findings.

Model	Cells/Material	Method	Main Findings	Reference
Spheroids	EEC16 and 12Z	polyHEMA-coated well plate	enhanced proangiogenic signaling in EEC16 3D culture	[17]
	12Z, St-T1b and ESC	hanging drop method	migration and matrix remodeling; behavior manipulation possible through external factors	[18]
	12Z and ESC	hanging drop method	urolithin affects spheroid integrity and reduces viability	[19]
	12Z, iEc-ESC, and iHUF	96-well micro-mold inserts	increased inflammation-associated gene expression; overlap with baboon gene expression; modulators influence invasion behavior	[20]
Organoids	primary eutopic and ectopic endometrial cells	Matrigel-containing droplets	severity level-dependent expression pattern	[21]
	primary eutopic and ectopic epithelial cells	droplets of liquefied growth factor-reduced Matrigel	alteration in epigenetic modification of HOX genes and cofactors	[22]
	primary eutopic and ectopic epithelial cells	droplets of liquefied growth factor-reduced Matrigel	disrupted progesterone signaling in endometriotic lesions	[22]
	primary eutopic and ectopic epithelial cells	Matrigel	responsiveness to estrogen and progesterone	[23]
Microfluidic systems	primary ESC and HPMC	static PDMS channels	peritoneal health affects stromal invasion behavior	[24]
	peritoneal fluid	droplet-based microfluidic platform	reduction in MMP-2 and ADAM-9 activities	[24]
	primary eutopic stromal cells	PDMS channels with flow	increased deformability and decreased stiffness	[25]
	12Z	microdevice with 3D lumen	increased intracellular oxygen radicals and reduced cell viability	[26]
Bioprinting	endometrial stromal and epithelial cells	extrusion-based bioprinting	cell viability, proliferation and ECM remodeling observed	[27]
	iPSC-derived endometrial-like cells	laser-assisted bioprinting	self-organization of endometrial structures and hormone responsiveness	[28]
CAM	primary endometrial stromal cells	grafted onto CAM of fertilized chicken eggs	increased vascularization and invasion in endometriotic-like lesions	[29]
	Patient-derived endometrial explants	CAM implantation	anti-angiogenic drug screening; reduction in lesion vascularization and stromal invasion	[[30], [31], [32]]
Explants	endometrial lesions from patients	ex vivo culture in Matrigel or collagen gels	cell proliferation, invasion, and fibrosis modeling	[33]
	mouse uterine explants	organ culture with estrogen and progesterone	progesterone resistance and inflammatory responses	[34]
	human ectopic endometrial tissue	explant xenografts in immune-deficient mice	lesion progression and response to targeted therapies	[30]

EEC16: ovarian endometriosis epithelial cell line EEC 16; 12Z: endometriosis cell line 12Z; polyHEMA: poly(2-hydroxyethyl methacrylate); 3D: 3 dimensional; ESC: endometrial stromal cells; iEc-ESC: immortalized endometrial cyst-endometriotic stromal cell line; iHUF: immortalized human uterine fibroblasts cell line; HOX: homebox; HPMC: peritoneal mesothelial cells; PDMS: polydimethylsiloxane; MMP: matrix metalloproteinase; ADAM: a disintegrin and metalloproteinase; ECM: extracellular matrix; iPSC: induced pluripotent stem cells; FRESH: Freeform Reversible Embedding; CAM: chorioallantoic membrane.

## 2 3D i*n vitro* models for studying endometriosis

### 2.1 Spheroids

Spheroids are scaffold-free systems, which can be built from a broad range of cells. The distinguishing characteristic is that spheroids are formed by self-aggregation of the cells, supported by the usage of appropriate culture plates or special physical parameters. The 3D structure closely mimics the cellular interactions, microenvironment and structural organization found in native tissues. In the context of endometriosis, especially epithelial and stromal cell lines are used to form spheroids. These models serve as a promising platform for studying cellular behavior, disease progression and the response to treatment strategies [20].

**Fig. 1:**
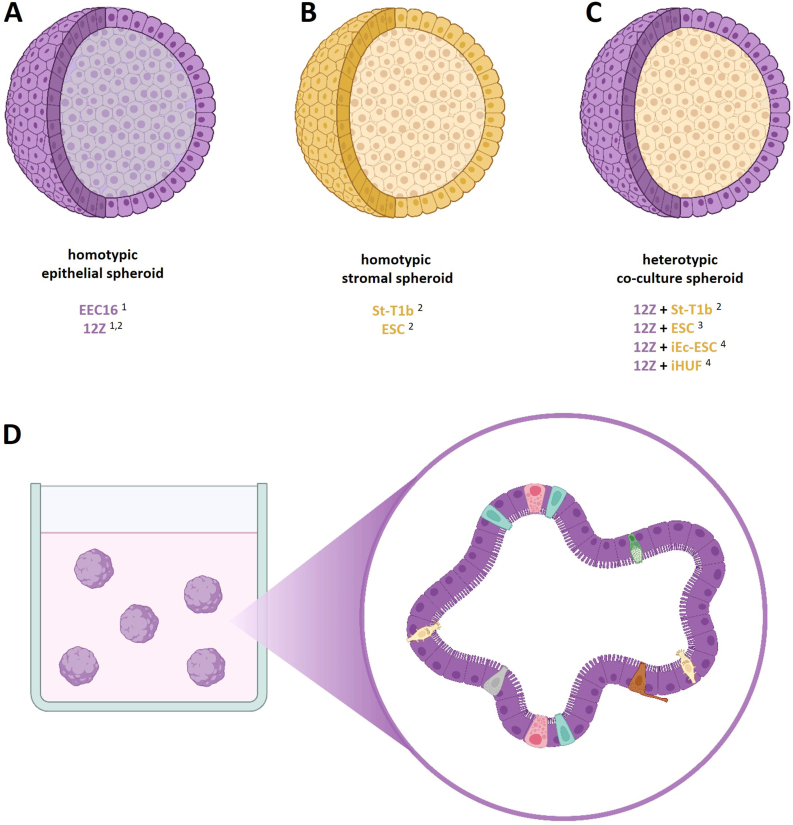
**Schematic representation of spheroids and organoids mimicking the environment of endometriotic lesions.** Homotypic spheroids are build up from a single cell line or primary cell, which can either be epithelial cells (A) or stromal cells (B). Heterotypic spheroids are co-culture models made up from epithelial cells, which form an outer shell, covering the stromal cell core (C). Different types of spheroids and co-culture models were developed as indicated. ^1^ [20]. ^2^ [17]. ^3^ [18]. ^4^ [35]. The organoids generated by Boretto et al. contained a lumen and a bordering cell layer (D). The microstructures include epithelial and secretory cells as well as cells with microvilli and cilia. Furthermore, luminal invasion, cell polarization and mucus secretion in the lumen were confirmed. EEC16: ovarian endometriosis epithelial cell line EEC 16; 12Z: endometriosis cell line 12Z; ESC: endometrial stromal cells; iEc-ESC: immortalized endometrial cyst-endometriotic stromal cell line; iHUF: immortalized human uterine fibroblasts cell line. Created in BioRender.com.

The first endometriosis spheroid model was developed by Brueggmann et al., in 2014. The group used a novel isolated ovarian endometriosis epithelial cell line (EEC16) and a well-established endometriosis cell line (12Z) to generate spheroids in a poly(2-hydroxyethyl methacrylate) (polyHEMA) coated well plate (Fig. 1A). Afterwards, they characterized the 3D model compared to 2D cell culture and human endometriosis lesions with the use of gene expression analysis and immunohistochemistry. Both spheroid types resemble the histological features of certain human endometriotic lesions. Furthermore, the authors analyzed the expression of genes involved in immune responses, local interactions and hormonal pathways. Both cell lines showed a highly similar trend in gene expression. However, when comparing 2D and 3D culture, significant alterations were observed in the expression level of certain genes, indicating enhanced proangiogenic signaling in EEC 3D culture. Based on these findings, Brueggmann et al. concluded that 3D spheroid models are a superior to traditional monolayer cultures for endometriosis modeling, offering a valuable opportunity to identify and implement new targeted treatment approaches [17].

Aiming to develop a model that mimics endometrial architecture and ectopic lesions, Stejskalová et al. utilized the hanging drop method to generate spheroids combined with either collagen I or Matrigel. They employed 12Z cells, the endometrial stromal cell line St-T1b - either in co-culture - or primary endometriotic endometrial stromal cells (ESC) (Fig. 1A–C). Notably, the 12Z spheroids and the co-culture spheroids were significantly larger than the St-T1b and ESC spheroids, despite individual 12Z cells being smaller than the other cell types. According to the cell proliferation assay, this is most likely attributed to the proliferation of 12Z cells within the spheroids. For St-T1b spheroids, they were able to demonstrate an altered expression of genes involved in migration and invasion. Matrix remodeling following directional migration was observed only in stromal spheroids, but not in single cells. They also showed a distinct invasive and migratory behavior of stromal spheroids in comparison to co-culture or 12Z spheroids, which was likewise more pronounced for all spheroids on collagen I than on Matrigel. This suggests an altered stromal invasiveness by interaction with epithelial cells [18]. According to previous research, this is most likely due to reduced MMP2 levels in co-cultured stromal and endothelial cells [35]. Additionally, they demonstrated various approaches to modulate invasion and migration behavior, including MMP or ROCK inhibitors as well as miRNAs. Thus, they established the spheroid model as a promising preclinical platform for evaluating small molecule- and micro-RNA-based drugs for endometriosis [18].

To examine the biological effects of botanical compounds on endometriosis, Mc Cormack et al. used a co-culture spheroid model of ESC and 12Z cells (Fig. 1C) for studying the effects of urolithin A and B, two plant-derived metabolites of ellagitannins formed by microbial degradation processes in the human gut. In addition to various effects observed in 2D cell culture, they demonstrated that urolithin treatment disrupts spheroid integrity and reduces viability. Given that a clinical trial with urolithin had not reported any negative side effects, the authors emphasize that the compounds provide a basis for further investigation, including *in vivo* studies [19].

A spheroid co-culture model existing of 12Z cells on the periphery and the immortalized endometrial cyst-endometriotic stromal cell line (iEc-ESC) or the healthy immortalized human uterine fibroblasts (iHUF) cell line as a core, was developed by Song et al., in 2023 (Fig. 1C). They formed the spheroids in 96-well micro-molds inserted into a 24-well plate. The group used this model to further investigate epithelial-stromal interactions and simulate peritoneal invasion involved in lesion formation. Through transcriptome analysis, they identified an increased expression of inflammation-associated genes including interleukins, prostaglandin synthase enzymes and telomerase reverse transcriptase in endometriotic spheroids. Furthermore, they determined a highly significant overlap of gene expression profiles in their endometriotic spheroids compared to endometriotic lesions especially from baboons but also from humans. Since cell invasion plays a crucial role in the initiation and development of endometriotic lesions, Song et al. designed a 3D invasion model. Therefore, they seeded endometriotic spheroids on top of human peritoneal mesothelial cells grown in Matrigel to mimic the peritoneum, exposing stromal cells as the leading edge of penetration and invasion. They also demonstrated altered invasion behavior in the presence of estradiol, progestin and pro-inflammatory macrophages. In doing so, the group established spheroids that effectively mimic numerous features of endometriotic lesions reinforcing spheroids as a valuable model for investigating the mechanisms underlying the formation of endometriotic lesions [20].

As the results show, endometriotic spheroids are particularly suitable to study migration and invasion processes. The simple structure allows to mimic the floating of menstrual reflux fragments as well as the initial attachment, the fragment compaction and the stromal-led invasion. With that, endometriotic organoids outperform other 3D models in investigating specific biological questions, such as: ‘How do multicellular endometrial aggregates adhere to and penetrate mesothelial and extracellular matrix barriers during early lesion establishment?’, ‘Which signaling pathways drive 3D invasion?’ and ‘Which cell–cell and cell–matrix adhesion molecules regulate the migration and invasive behavior of endometriotic cell clusters?’. Besides that, the incorporation of endometriotic cell lines in spheroid formation enables simple handling along with high cost-effectiveness and allows convenient access, ensures biological material stability and enhances experimental reproducibility. The mentioned models show that spheroid culturing allows to follow cellular processes such as hormonal signaling, cell-cell interactions, growth factor regulation and neovascularization pathways. With this, spheroids were established as an endometriosis model surpassing traditional monolayer culture and serve as a capable *in vitro* model for the discovery and development of targeted therapeutic approaches [14].

### 2.2 Organoids

Just like spheroids, organoids are also artificially *in vitro* generated self-organizing 3D structures. But in contrast, embryonic stem cells, induced pluripotent stem cells or cells isolated from tissue are used to form the complex clusters. In many cases, organoids are scaffold-based systems, which means, that natural or synthetic materials support the self-aggregation of the organ specific cells. Besides that, embryoid bodies are another type of organoid systems. The formation occurs independently of Matrigel or other natural or synthetic scaffold materials. The 3D structure is formed by pluripotent stem cells, such as embryonic stem cells or induced pluripotent stem cells, relying solely on intrinsic cell–cell interactions and self-organizing mechanisms. Embryoid bodies are used to mimicking early embryonic tissues, since the cells are capable to differentiate into the cell types of all three germ layers [36]. Nevertheless, with respect to endometriosis, the published organoid models are scaffold-reliant systems based on primary cells from endometriotic lesions. These models are a powerful tool to study various biological and pathological characteristics of the organ of origin, since the native physiology and functional features are well-represented [37,38].

In 2019, Boretto et al. generated the first patient-derived endometriotic organoids, by cell cultivation in droplets mainly consisting of Matrigel. They created those long-term expandable organ models based on healthy endometrium or manifold endometrial pathologies, among others from endometriotic lesions of eutopic and all ectopic implantation sites as well as all severity stages (Fig. 1D). The ectopic organoids reflected several phenotypic characteristics as found in endometriotic lesions, such as mucus secretion, polarization and luminal invasion. Furthermore, the authors demonstrated the expression of endometriotic markers after the implantation of ectopic organoids into the peritoneal cavity of NOD-SCID mice. By genetic and transcriptomic analyses, they showed alterations in several pathways in ectopic organoids, including PI3K-AKT, WNT and Hippo signaling as well as hormonal, adhesion and invasion pathways. Likewise, they observed a severity level-dependent expression pattern of various genes. With that, Boretto et al. provide a valuable model for endometriosis research as well as a tool for drug discovery and screening [21].

Organoids were also used to analyze the DNA methylation of human homebox (HOX) genes and the corresponding cofactors. Esfandiari et al. established an organoid model, consisting of epithelial cells from eutopic and ectopic endometriotic tissue, by cultivation in droplets of liquefied growth factor reduced Matrigel. They compared the methylation patterns of HOX genes A-D and cofactors in control endometrium, biopsy specimens and organoids from both, eutopic and ectopic tissue. On the one hand, they showed methylation alterations in eutopic and ectopic biopsies and organoids in comparison to the control tissue. Additionally, the methylation pattern differs in eutopic endometrium and ectopic lesions. Two thirds (56 of 84) of the investigated genes displayed similar patterns in eutopic biopsies and eutopic organoids, and also ectopic biopsies and ectopic organoids followed the same trend. Thus, the authors concluded that the epigenetic changes are preserved in the endometriosis organoids and picture those as a suitable model to study the epigenetic mechanisms of endometriosis [39].

Since progesterone resistance is a known phenomenon in endometriosis, the group of Esfandiari et al. used the previously described model to examine the responsiveness of endometriosis organoids to progesterone and analyzed molecular-level disruptions in progesterone receptor isoform B (PRB) pathway. Besides gene expression analysis of endometriosis-related genes, they evaluated the expression of progesterone-regulated genes such as 17HSDβ2, PAEP and LIF compared to control organoids. Their results correlate with implantation failure, a condition commonly observed in clinical cases of endometriosis. Furthermore, they demonstrated a significantly decreased PRB mRNA level in both, eutopic and ectopic organoids, compared to control. What is unusual, however, is that this was accompanied by a hypermethylation of the PRB promoter in eutopic organoids, but not in ectopic ones. This suggests other epigenetic mechanisms for transcription suppression, such as microRNAs and histone modifications, taking effect in ectopic organoids. Overall, Esfandiari et al. established organoids, which serve as effective preclinical models for unraveling the mechanisms behind disrupted progesterone signaling in endometriosis [39].

Another organoid model was developed in 2025 by Zhang et al. comprising epithelial cells from ectopic ovarian lesions or eutopic endometrial tissue from patients with endometriosis. The organoids were created by embedding the cells in Matrigel. The authors successfully reproduced the features of native endometrial tissue, including secretory ability, proliferation capability and typical glandular arrangements. A concentration-dependent effect on the epithelial organoids was demonstrated by treatment with estrogen and progesterone, resulting in enhanced or inhibited growth, respectively. With this model, Zhang et al. developed two types of endometriosis organoids, which exhibit essential phenotypic and functional characteristics of the disease, and thus, offer a novel 3D *in vitro* model for investigating drug interventions and the underlying mechanisms of ovarian endometriosis [23].

As demonstrated by the established models, organoids have a clear advantage over other models in studying implantation, hormone-pathways and -responsiveness or epigenetics. The complex structure, differentiation, glandular function and especially the disease heterogeneity enable a wide variety of endometriosis-related issues to be investigated, such as the regulation of epithelial function in endometriotic organoids by estrogen and progesterone or the influence of genetic diversity on hormone responsiveness and drug sensitivity. Furthermore, in organoids, the use of human-derived endometriotic cells enables the study of disease characteristics across different clinical stages and lesion localizations. In this context, organoids serve as a model that closely replicates the pathomechanisms of endometriosis, capturing its heterogeneity and accurately representing the disease genotype [14].

### 2.3 Microfluidic organ-on-a-chip models

Microfluidic organ-on-a-chip models are engineered miniature systems, that reproduce the physiology of an organ *in vitro*, including its key structure and features. Living cells are cultured in microchannels with a precisely regulated fluid flow, nutrient exchange and mechanical force, which simulate the physiological activity and response of an entire organ. In endometriosis research, primary cells, cell lines as well as body fluids are used in distinct organ-on-a-chip models. These systems provide a highly controlled and dynamic platform, to study diverse aspects of endometriosis such as the complex physiological environment, functionalities, cellular interactions and disease mechanisms in tissues [40,41].

**Fig. 2:**
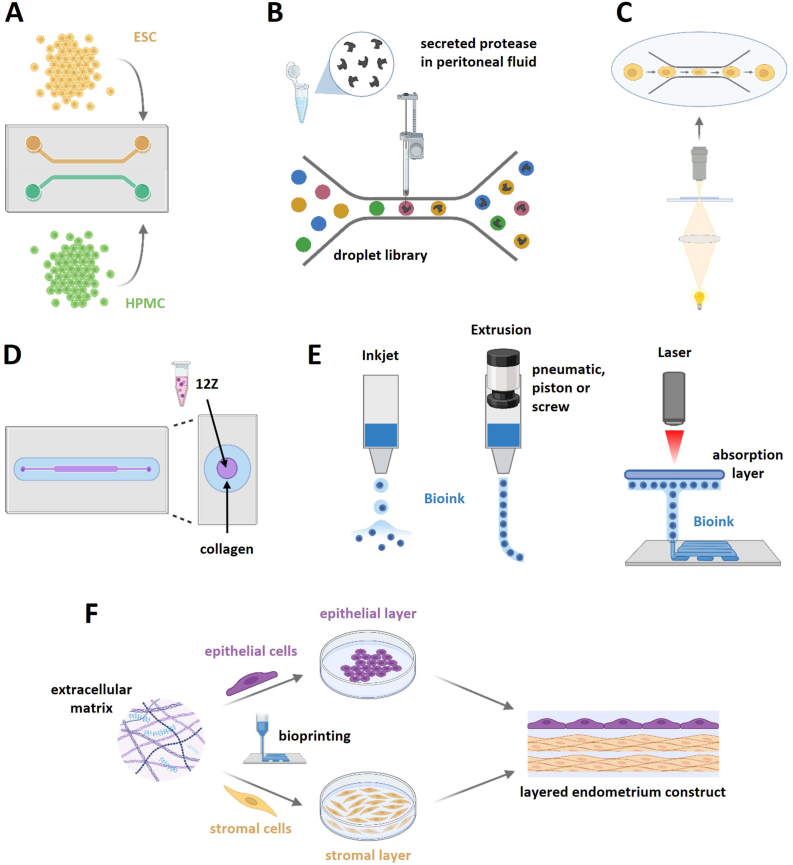
**An illustration of organ-on-a-chip and bioprinting models that simulate the endometriotic microenvironment**. (A) Healthy and diseased primary endometrial stromal cells (ESC) and peritoneal mesothelial cells (HPMC) are co-cultured in two adjacent static polydimethylsiloxane channels, which are removable. (B) Samples of secreted proteases from peritoneal fluid from patients with and without endometriosis are injected into a barcoded droplet library of protease substrates and inhibitors. (C) Deformation index and cell velocity of endometrial and endometriotic stromal cells are imaged during their flow through a microfluidic platform of polydimethylsiloxane microchannels. (D) The endometriosis cell line 12Z is inoculated in a luminal microdevice with collagen hydrogel. (E) Different technologies are available for bioprinting, comprising inkjet-based, extrusion-based and laser-based bioprinting strategies. (F) Schematic representation of a 3D bioprinting application in endometrial modeling. Created in BioRender.com.

In 2011, Chen et al. published a static, polydimethylsiloxane (PDMS)-channel-based model to simulate the endometriosis environment (Fig. 2A). Those chips included primary ESCs and peritoneal mesothelial cells (HPMCs) in a healthy or diseased stage. The cells were co-cultured in two adjacent channels, which were later removed to dynamically study cell-cell-interactions and migration behavior. The results showed that HPMCs in the control group resisted stromal cell invasion in both, pathological and healthy state. In contrast, both types of stromal cells successfully invaded diseased HPMCs. By analyzing the interactions between stromal and mesothelial cells, the group demonstrated reduced adhesion between endometriotic HPMCs, followed by apoptosis after stromal cell invasion. Based on these findings, they concluded that peritoneal health plays a crucial role in the establishment of endometriosis [22].

Another group around Chen et al. developed a droplet-based microfluidic platform, which allows a high-scale tracking by the injection of samples into a barcoded droplet library, containing multiple mixtures of protease substrates and inhibitors (Fig. 2B). They utilized the method on peritoneal fluid samples from individuals with and without endometriosis. Thereby, they discovered relevant physiological differences between healthy and diseased tissue, notably a reduction in MMP-2 and ADAM-9 activities. The observed decline of metalloproteinase activity in endometriotic tissue helps to explain the clinical phenomenon of endometriotic cysts remaining non-invasive to the surrounding tissue. Through the study, the authors developed a versatile and highly customizable tool, which can be adapted for various applications, such as non-invasive diagnostic screening or assessment of the inhibitory activity of a sample with a droplet library containing spiked-in recombinant enzymes [22].

To characterize the mechanical properties of eutopic endometriotic stromal cells, Altayyeb et al. established a microfluidic platform of PDMS microchannels with a constant flow rate (Fig. 2C). The flow within the channels was imaged to track the deformation index and cell velocity, providing insides for their cellular stiffness. They found that endometriotic stromal cells exhibited increased deformability and reduced stiffness compared to healthy stromal cells. Using their method, the group proposed biomechanical properties of endometriotic stromal cells as a potential basis for identifying a mechanical biomarker for endometriosis [25].

A luminal organ-on-a-chip model for endometriosis was provided by Kapur et al., in 2023. They built a microdevice with a 3D lumen in a collagen hydrogel and inoculated 12Z cells (Fig. 2D). They used the model to test the oxidative phosphorylation inhibitors curcumin, plumbagin and atovaquone against the endometriosis cell line. For each substance, they observed a reduced cell viability by inhibition of proliferation and initiation of cell death. Furthermore, they measured an increased intracellular level of oxygen radicals and DNA double strand breaks. Based on these results, they highlighted oxidative phosphorylation inhibitors as an innovative therapeutic approach for endometriosis [26].

Overall, the organ-on-a-chip platforms combine the possibility to mimic the tissue microenvironment with a controllable fluid dynamic, which allows to capture the complexity of tissue and its supply. The microfluidic system offers benefits such as cost-effectiveness due to minimal sample requirements, high resolution, rapid analysis and enhanced sensitivity [14]. Additionally, the capability for a high-throughput setup, establishes the model as a favorable approach for drug screening, disease mechanism studies and biomarker discovery.

### 2.4 Bioprinting

3D bioprinting is a computer-assisted technology used to design a print that incorporates specific cells or tissues to create biologically engineered structures. This technique has emerged as a flexible and important tool in tissue engineering of functional 3D biostructures with better geometric designs to bridge the divergence between engineered and natural tissue constructs. Despite small-scale applications of the model in clinical settings due to high cost and sometimes poor mimicking, this *in vitro* 3D model gives a revolutionary approach in fabricating customized tissues.

The approaches used in in vitro 3D bioprinting are classified based on the strategy and complexity (Fig. 4). Extrusion-based bioprinting has been widely used in creating 3D tissues. The bioink is extruded from the printhead using mechanical pressure, pneumatic pressure, or screw, and stacked layer by layer according to a preset path that forms a three-dimensional structure. On the other hand, the Laser-assisted bioprinting uses high-energy laser pulses

to generate shock waves on the surface of bioink, pushing the ink to form tiny droplets or microfilaments, which are accurately deposited to the target location to achieve the construction of three-dimensional structures [42]. Inkjet-based bioprinting, also known as drop-on-demand bioprinting, evolves from commercial two-dimensional (2D) inkjet printers. This technique sprays bioinks at designated positions in form of tiny droplets through a hot air bubble, piezoelectric effects, and stacked layer by layer to form a three-dimensional structure [42]. The 3D bioprinting is basically composed of three parts: hardware, software, and material. Therefore, the biomaterials used in bioinks formulations should maintain the correct architecture for the mimicry of the tissue of interest [42]. Several hydrogels have been used to fuse cells of the endometrial origin to create endometrial tissues despite having very few applications of this technique in endometriosis studies.

Nie et al. described a hydrogel composed of sodium alginate and hyaluronic acid to fabricate an endometrial construct. Ionic cross-linking of the alginate network with Ca^2+^ ions to achieve robust gel formationresulted in high cell viability and supported the restoration of organized glandular structures in the engineered tissue. The low molecular weight hyaluronans enhanced bioactivity [43,44]. Their findings indicate restored morphology and structure of the uterine wall with enhanced vascularization. The findings were validated in *in vivo* conditions indicating the possibility of using the model in studying endometriosis by inducing disease factors.

Suitable bioink rigidity and porosity are needed for structural maintenance and cell growth, respectively. The correct rigidity and porosity vary from tissue to tissue. For example, the rigidity of the soft tissues is approximately 0.2–5 kPa, whereas with hard tissues up to 15,000 kPa Therefore, endometriosis-targeted 3D bioprinting revolves on the interplay between bioink formulation, mechanical performance, and biological function, since each domain critically shapes the fidelity of *in vitro* disease models. The type of endometriosis is a determinant in matrix remodelling [45].

Deep infiltrating endometriosis is characterized by dense and fibrous tissue, a hallmark of fibrosis. Therefore, the bioink characteristics will be defined by the use of stiff substrates in *In vitro* experiments to mimic fibrosis [46]. However, a caveat is associated with these matrices, because several studies indicate that stiff matrices yield high stress. These changes in the stiffness determined by the bioink composition has an effect on the behaviour of endometriotic cells. For example, soft matrices have been reported to have an inhibitory effect on cell proliferation [47].

The rheological properties of bioinks, such as viscosity, determine the printability and structural fidelity. Cell-laden hydrogels can be deposited into stable, complex architectures without compromising cell viability or resolution. [48]. However, an increase of the polymer concentration or crosslinking achieves higher mechanical stiffness commonly increases viscosity and yield stress [27]. Mechanical cues from the extracellular matrix (ECM) regulate vascular endothelial cell (EC) morphology and function. Since naturally-derived ECMs are viscoelastic, cells respond to viscoelastic matrices that exhibit stress relaxation, in which a cell-applied force results in matrix remodeling. Natural polymers such as collagen I have been widely used to offer the desired characteristics of the specific modelling question. However, synthetic polymers e.g polyethylene glycol (PEG) can also be used to offer the desired mechanical properties. For instance, the 3D model used by Yi et al., 2025 used low doses of genipin (0.5 mM) to stabilize the collagen in the 3D equine model. This model was composed of endometrial stroma and epithelial layers. This biomaterial achieved the desired biological print characteristics and at the same time improved cell viability. [49].

Notably, the human endometrium undergoes cyclical remodeling that affects the mechanical properties which modulates the cell behavior. Cells sense these shifts in the stiffness of their extracellular environment via mechanosensitive pathways such as YAP/TAZ signaling [22].

From a mechanobiological perspective, human endometrial tissue shows menstrual-cycle dependent alterations in stiffness and viscosities. The viscoelastic properties of the endometrium vary across the menstrual cycle: tissue rigidity and associated rheological parameters are generally highest during the proliferative phase, decrease to intermediate levels in the secretory phase, and reach their lowest values following menstrual shedding [50]. In *in vitro* 3D bioprinting, the disease biology strongly motivates more stiff and fibrous-like matrices models applied in endometriosis studies, which can demand a higher extrusion pressure. However, increasing the extrusion pressure increases the shear stresses that threatens the viability of encapsulated cells, particularly when mimicking lesions of deep endometriosis. Therefore, the bioink formulation needs to include menstrual phase-appropriate polymers for example higher crosslinking GELMA or ECM for proliferation [50]. The use of gelatin methacryloyl (GelMA) in bioprinting 3D *in vitro* models is driven by biocompatibility and mechanical properties allowing for accurate bio-fabrication of structurally consistent hydrogels [51]. The use of GelMA in the 3D endometrial *in vitro* models is customized by adjusting variables such as weight, light exposure, and concentration. The desired viscosity can be achieved by adding other molecules such as fibronectin [52].

Cook et al. employed a synthetic polyethylene macromer functionalized with vinyl sulfone to encapsulate stromal cells and later overlayed epithelial cells. Polyethylene glycol (PEG) is synthetic polymer with the properties of high compatibility, less prone to non-immunogenic degradation, and easily modified. The Incorporation of adhesives and ECM-binding peptides that target ECM promoted enhanced basement membrane deposition beneath the epithelial layer and increased collagen assembly by stromal cells. This modular PEG hydrogel supports physiologically relevant, long-term co-cultures of epithelial and stromal cells reminiscent of mucosal barrier tissue, enabling dynamic cell-matrix interactions and tissue-like organization *in vitro* [53].

Decellularized ECM (dECM) bioinks are derived from natural tissues but they retain the important biochemical cues, structural proteins and growth factors that are important for tissue specific functionality. dECMs have been used in endometrial regeneration for example supporting the epithelial stromal interactions essential for tissue repair and implantation [50]. Wen et al. designed a 3D-bioprinted model aiming to create a customized 3D-printed hydrogel scaffold delivering granulocyte colony-stimulating factor (G-CSF) to endometrial regeneration after severe uterine injury. This was enriched with granulocyte colony-stimulating factor loaded sustained-release microsphere (G-CSF)- (G-CSF-SRM) hydrogel scaffold to effectively promote the regeneration of the endometrium. These findings highlight the potential to bioprint the endometriotic cells for functional studies *in vitro.* We note that endometriosis has similar features with an injured endometrium. The approach by Wen et al. could provide a module that can be adapted for endometriosis 3D modelling, for instance, the use of the stromal and epithelial cells on scaffolds containing endometriosis-related factors [54].

Despite sparse information on the application of 3D bioprinting in endometriosis, a scaffold-free based biofabrication model has been established to bypass hydrogel rheology tradition. Wendel's study on the scaffold-free spheroids-based bioprinting model is a precise example for *in vitro 3D bioprinting* endometriosis model that demonstrates the feasibility of constructing a lesion-like tissue from patient cells without using a scaffold material. In this study, spheroids approx. 500uM using the endometriotic cell line 12Z mixed with the endometrial stromal cell (T-HESC) using the Regenova Bio 3D printer. The Kenzan device has needles where the spheroids are screwed in a 3D arrangement, and over a few days the spheroids fuse with the adjacent spheroids to form a continuous tissue. [52]. This technique has achieved significant progress in capturing the complexity of various tissues, however we recognize the challenging processes for example optimization and other experimental factors to achieve a suitable printing technique and biomaterials, cell growth, the biological environment, microarchitecture, and the functionality of the tissue.

### 2.5 Chicken Chorioallantoic Membranes and amniotic membrane cultures

**Fig. 3:**
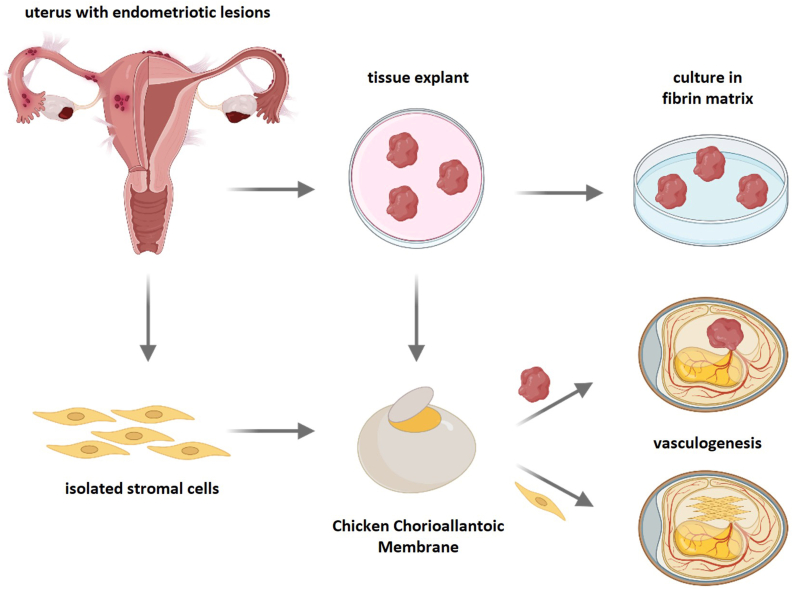
**Scheme of Chicken Chorioallantoic Membranes and explants to model endometriosis**. Endometrial explants and stromal cells are isolated from endometriotic lesions. Explants can be embedded in a fibrin matrix or, similar to stromal cells, directly seeded onto the chicken chorioallantoic membrane (CAM). This *in vivo* platform allows the study of vasculogenic and angiogenic processes relevant to endometriosis pathophysiology.

Chicken Chorioallantoic Membranes (CAMs) and amniotic membrane (AMs) are considered an alternative to *in vivo* and ex vivo study systems, respectively. Both models are deemed to be simple, high reproducibility, and cost-effective. These models contain an extraembryonic tissue layers formed by the fusion of the chorion with the vascularized allantoic membrane making them characteristically abundant of collagen IV, fibronectin, and other ECM components, which play essential roles in blood vessel formation (angiogenesis) (Fig. 3). Despite their widespread application in cancer and tumor research, CAMs and AMs have not been extensively utilized in endometriosis studies. Nevertheless, these models offer hope for bridging the gap between cell-based and animal-based assays, such as the use of endometriosis patient-derived xenografts (PDX) to gain practical insight into their translational potential [14,30,55].

**Fig. 4:**
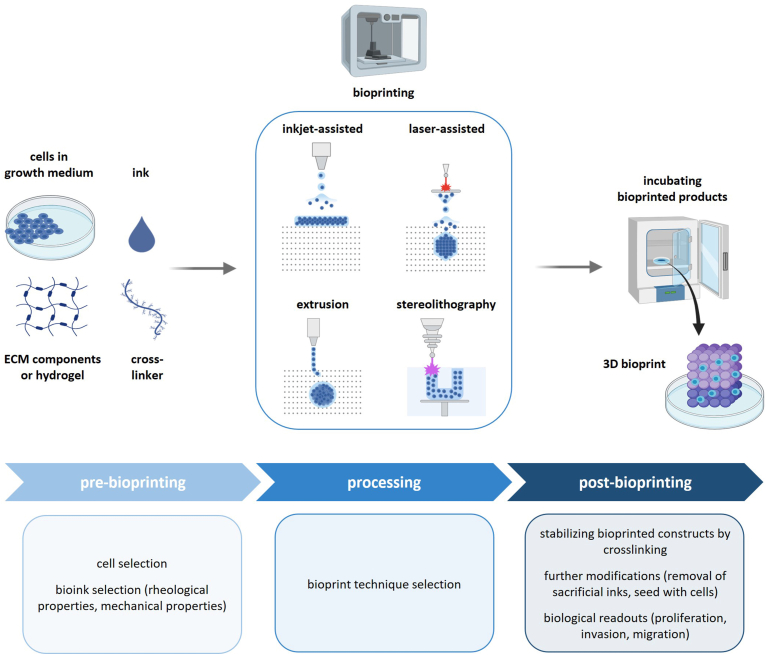
**Strategy for bioprinting of endometriotic tissue**. The pre-bioprinting phase includes the proper choice of cell types, growth media, the composition (selected ECM components of hydrogel) as well as the proper crosslinker which enables the modulation of the stiffness of the 3D bioprinted tissue (left panel). Different processing technologies determine the resolution and desired architecture of the bioprinted tissue and can be adapted to differences in the sensitivity of different cell types to mechanical stress (center). The post-bioprinting phase allows for modification of the bioprinted matrix (e.g. by crosslinking under different experimental conditions), and adaptation of the cell culture conditions to relevant research questions in endometriosis research, e.g. the study of proliferation, migration and invasion.

The AMs have been applied in studies involving the interactions between endometrial cells and ECM to help evaluate the expression of cell adhesion molecules in the endometrial cells in which the studies are important in understanding the pathogenesis of endometriosis [56]. This study by van der Linden et al. (1998) shows that the AMs epithelium layer overlies a loose avascular stroma that consists of Collagen and Elastin elaborating the significance of the model in combining functional studies with structural studies [56].

The review article by Juhasz-Böss et al. provides in-depth insight into the chorioallantoic membrane (CAM) model in eggs from avian species. This review elucidates the usefulness of CAM models for quantitative studies comparing vascularization, drug permeability, and tissue viability across several species [57]. In addition, the CAM models can significantly contribute to studies on extracellular vesicles and regenerative medicine [29].

Nap et al.'s research on angiogenesis, a recognized characteristic of endometriosis, clarifies that its inhibition may serve as a promising therapeutic strategy. They appropriately cite evidence of angiogenesis in endometriotic lesions and eutopic endometrium of patients, supporting the choice to test angiostatin agents. They discuss the applications of the chicken embryo in studying tissue development, tumor growth, angiogenesis, and drug deliver [58]. They also highlight the model's ethical advantages and its utility in integrating human tumor cells for cancer research, which can also be beneficial for studies on endometriosis.

The research conducted by Juhasz-Böss et al. investigates the expression of matrix metalloproteinases 1 and 2 (MMP-1 and MMP-2). They employed this model to study mRNA expression levels of MMP-1 and MMP-2 at various time points. This model has proven valuable in enhancing our understanding of gene expression patterns during the early stages of lesion formation. This study elucidates that CAM models provide a unique *in vivo*-like environment to explore the behavior of endometrial tissue, particularly in relation understanding the invasive properties of endometriotic lesions.

Furthermore, the CAM model is a valuable intermediary between *in vitro* assays and mammalian *in vivo* studies. This is because of their dense vascular network, which provides an optimal environment for evaluating nano-bio interactions. This feature is particularly critical for assessing the therapeutic potential of nanoparticles in early diagnostics and gene therapy, where targeted angiogenesis plays a key role [59].

### 2.6 Explants

Explants are comprised of tissues sourced from the mother tissue, which is then transplanted into another vessel or cultured *in vitro*. These pieces of tissue serve as fundamental material for both *in vitro* and *in vivo* cultures. These explant models are extensively used to evaluate tissue viability in clinical trials that involve targeted treatments [30].

Endometrial explants have been vital in reproductive health research. For instance, explant culture models have been employed to examine the significant effects of external test compounds on gene expression biomarkers and studies involving the regeneration of endometrial-like tissues [30,60]. Researchers view explants as simple models that accurately replicate the endometrium both *in vivo* and *in vitro* systems. The tissues, derived from the parent endometrium, are acknowledged for their ability to imitate the early stages of endometriosis, yielding valuable information on tissue proliferation, angiogenesis, and invasion.

Fasciani et al. (2003) showed that endometrial explants can grow and invade a fibrin matrix, leading to the formation of glands, stroma, and endometrial vessels. They suggested that the growth and proliferation of endometrial tissue explants depend on the activation of vasculogenesis to supply necessary nourishment for the tissue's survival. The emergence of blood vessels or vascularization that originates from the endometrial tissue, indicates that the growth of the explant is inherent to the endometrial fragment [60,61]. Likewise, Fasciani et al. found that peritoneal fluid from both endometriosis patients and women without endometriosis had comparable effects on the proliferation of eutopic endometrial epithelial and stromal cells *in vitro*, also the induction of the expression of VEGF-A, which underscores its significance in cellular proliferation and angiogenesis [60].

Endometrial samples from the fundus of the uterine cavity have been cultured in a 3D fibrin matrix system to demonstrate significant biological activities, including the study of matrix metalloproteinase activity, angiogenesis, an increase in size, and implantation capability of blood vessels [62]. This has demonstrated the viability and the promise of using explants despite the challenges in understanding the progression of endometriosis. With their simplistic nature and being short-term models, the explants have been termed as the appropriate models for studying the early stages of endometriosis. The culturing of the piece of tissue *in vitro* and monitoring its biological activities can be done on both a normal endometrial explant and an endometriosis tissue explant [62].

Explant models are continuously evolving and can be readily adapted to *in vivo* conditions, proving valuable for ultrastructural organization research. In studies conducted by Franc Es-Herrero et al.*,* a 3D model of hetero-spheroids was developed using a bovine micro-sphere endometrial explant to investigate the hormonal responses *in vivo* as well as the expression of MMP [63].

In their experiments, Schäfer et al. examined gene expression alterations resulting from chemical and hormonal treatments using explants and demonstrating their utility for ex vivo studies. Additionally, endometrial explants have been employed to explore the ultrastructural organization of endometrial outgrowths and the impact of dienogest, a progestin highly specific to progesterone receptors [30]. A notable characteristic of this model is its cellular heterogeneity, which supports the notion that growth in a 3D fibrin matrix resembles the initial stages of endometriosis. Notably, the angiogenic factors COX-2 and glycodelin have been detected in this model, confirming its suitability for angiogenesis studies [58,60,64].

In conclusion, three-dimensional explant models offer a highly physiologically relevant experimental platform for studying endometriosis because they preserve native tissue architecture and enable integrated functional assessments of key pathological processes such as tissue invasion, hormone responsiveness, and immune-mediated interactions [60].

## 3 Limitations and current challenges

3D *in vitro* models have proven to be versatile and important tools for the investigation of essential cellular and molecular endometriosis mechanisms. Nevertheless, each model has certain limitations and challenges, which also need to be critically examined (Table 2).

**Table 2:** Summary of 3D In vitro and ex vivo models used in endometriosis research: Materials, methods, strengths, and challenges.

Model	Material	Method	Strengths	Challenges
Spheroids	cell lines	scaffold-free, self-assembling cell aggregates	simple, cost-effective, convenient access, stable biological material and enhanced experimental reproducibility	restricted range of phenotypes, limited microenvironment complexity and lack of vascularization
Organoid	primary cells	scaffold-based, self-organizing micro-structures	patient-like conditions, mimics heterogeneity and disease characteristics of different stages and localizations	limited uniformity and reproducibility, lack of perfusion and higher costs
Microfluidic systems	cell lines and primary material	controllable fluid dynamic	cost-effective, minimal sample requirement, high resolution, fast, sensitive, high-throughput compatible	complexity requires precision, small sample quantities may hamper analysis, high initial costs and long setup times
Bioprinting	cell lines, primary cells and spheroids	embedding in bio-inks	high spatial control and reproducibility, simulates cellular interactions and native microenvironment	high complexity, time-consuming and expensive optimization, lacks vascularization, limited long-term culture
CAM	primary material	grafted on biological membranes	cost-effective, allows vascularization and implantation studies	altered expression of growth factors and adhesion molecules, short experimental window, high variability, ethical challenge
Explants	primary material	ex vivo cultivation	maintains native tissue architecture and cellular interactions	limited long-term viability, low scalability and high-throughput potential, high variability

Spheroids are formed from different cell lines, which on the one hand results in a simple and cost-effective method, but on the other hand reflects only a restricted range of phenotypes and with that, impedes the depiction of the complex endometriosis nature [18]. Additionally, the limited microenvironment complexity represents a disadvantage of the model, including the lack of dynamic interactions with immune cells and the peritoneal or endometrial tissue environment as well as the systemic hormonal cycles [20]. Likewise, endometriosis spheroids usually miss the integration of blood vessels and thus investigation of angiogenesis, vasculogenesis, nutrient gradients or hypoxia, which are key processes in the lesion formation and progression *in vivo*.

Primary cells from endometriotic lesions are used for the formation of organoid models, which better represent the multifaceted character and heterogeneity of endometriosis. Simultaneously, this limits the uniformity and reproducibility of organoids, which is a significant obstacle for their usage in high-throughput screening and drug testing [65]. Additionally, the usage of primary cells leads to higher costs due to the necessity to use defined growth factor supplements, and a more time-consuming handling, as the cells need to be purified from a biopsy and characterized for proper marker expression. Another problem occurring in organoids is the lack of perfusion, which creates challenges in delivering nutrients, facilitating gas exchange and removing waste from the core. Moreover, many endometriotic organoids are mainly built from primary epithelial cells, but lack the stromal component, which limits the capability of the model as a replica of the endometriotic niche [66,67].

Microfluidic systems allow to culture endometriotic cell lines as well as primary material in a controllable fluid dynamic system with potential for the usage as a high-throughput platform. However, this is accompanied by high technical complexity and requirement for specialized equipment, expertise and precise fabrication. Although microfluidic systems count as cost-effectiveness due to minimal sample requirements, developing and optimizing often involves higher initial costs and longer setup times. On the other hand, the small sample quantities can lead to several challenges in further analysis [68,69].

Bioprinting enables high spatial control and reproducibility, while simulating cellular interactions and native microenvironment of endometriosis [14]. The greatest challenge in bioprinting is probably the complexity of the technique. The establishment of an appropriate model requires huge expertise in technical handling as well as time-consuming optimization processes and leads to high costs [70]. Besides that, bioprinting endometriosis models lack vascularization, which results in time constraints regarding long-term culture due to a hampered nutrient diffusion, especially in larger constructs [71].

The native tissue architecture and cellular interactions of endometriotic lesions are maintained in explant models, but nevertheless, once explanted, the tissue loses key systemic influences, such as hormonal cycles and immune responses, which are critical for modeling endometriosis accurately [62]. Due to their size, the nutrient supply is a critical factor in explant culture, resulting in a limited long-term viability. Moreover, the complexity and reliance on fresh endometriotic tissue limits the scalability and high-throughput potential of the model. Depending on the location, the explants suffer from mixed cell populations and, in addition, significant biological variation between tissue donors can affect experimental consistency and reproducibility [30,60,62].

CAM is a cost-effective model, suitable for studying endometriotic lesion implantation and angiogenic processes. However, the tissue's embryological origin results in an altered expression of growth factors, ECM regulators and adhesion molecules [72]. Additionally, native material causes considerable challenges in use due to its fast biodegradation, poor biomechanical consistency and the need for careful storage techniques [14,73]. Other constraints of the CAM model are the short experimental window, due to rapid embryo development, as well as the high variability in embryo development, vessel density and membrane morphology [74].

## 4 Future directions and prospects

The incomplete understanding of the pathogenesis of endometriosis hinders successful diagnosis and treatment. It is believed that new approaches, such as genetic testing, drug repurposing, and more personalized methods for symptom management, will help in reframing endometriosis. This review highlights the key 3D *in vitro* models and their unique strengths in modelling the endometrium. Nevertheless, none of the models has been perfect in mimicking the complexities of the endometrium. Therefore, future progressive efforts are necessary to further increase the physiological relevance of the models [75].

3D *in vitro* bioprinting technology is versatile and allows for the integration of different models. For example, it can be applied together with micro-fluidics to print an endometrial lesion perfused with simulated peritoneal fluid under controlled conditions. The integration approach will improve the applicability of the model especially for drug testing. The 3D *in vitro* bioprinting technology has made substantial progress by creating bilayers of endometrial stromal and epithelial cells. However, we highlight the need to include other cell types for example the immune and endothelial cells. Moreover, regular advances in biomaterial properties and printing resolution (e.g by using photolithography-based systems) are necessary to improve the fidelity of the constructs [71].

Organ on a chip (OOC) systems are high-throughput *in vitro* models that combine organoid technology and microfluidic chips. The OOC model has been popular in different research areas, however, so far applications in endometrial and endometriosis studies are limited. For instance, the technology has been applied in mimicking the functional alveolar capillary interface of the human lungs, demonstrating that modeling of complex physiological environments is possible using this approach [76]. This extensive use of this technology and development of more advanced biomaterials that meet the specific cellular, ECM and endocrine requirements of the endometrium will optimize the system to enable construction of the correct architecture of endometriosis in *vitro* [77].

Organoids are important 3D *in vitro* models that are mainly developed from epithelial cells from the various endometriotic tissues. Organoid modelling is promising in mimicking the native features of the endometrial tissue [23]. An important direction of this model could be the development of a co-culture organoid system that incorporates the stromal cells, immune cells, and the endothelial cells to culture an endometriotic tissue [78]. I addition, there is need to establish organoids from various lesion subtypes including the superficial peritoneal and deep infiltration nodules to allow set up biobanks that capture the heterogeneity of the endometriosis.

We discuss various 3D models each displaying different strengths. We foresee a future of hybrid models using their strengths to broaden the application. For instance, spheroid 3D *in vitro* models are very powerful in studying invasive behavior of cells as discussed in this review [18]. On the other hand, the classical CAM models are well utilized in angiogenesis-related studies. Creating a hybrid model of spheroids together with the classical CAM model will help to study how pre-formed spheroids vascularise and provide endometriotic lesions with nutrients [52].

Integrating CRISPR (Clustered Regularly Interspaced Short Palindromic Repeats) into *in vitro* 3D models will be beneficial for gene manipulations in *in vitro* conditions before it could be applied to *in vivo* models [75,79]. Although this technique has been around for some time, it has only recently gained prominence in endometrial and endometriosis research, particularly with studies involving gene expression, gene knock-out, and knockdown. Wang et al. have used the CRISPR/Cas9 system to knock out the AT-rich interactive domain 1A gene in the Ishikawa cell line while investigating progesterone resistance in endometrial cancer. For clinical and therapeutic applications, this technology can be used to evaluate cell behaviour in a 3D *in vitro* environment using the models discussed. The future lies in the incorporation of this manipulations to study the gene functions and understanding the multicellular complexities of endometriosis, for instance, the invasive or pro-inflammatory cell behaviors that are seen *in vivo* [52]. In hormonal response studies, CRISPR/Cas9 can be efficiently used to edit and correct a specific mutation on organoids derived from endometriosis. This could be a bridge towards studies involving the hormonal pathways, and serve as a powerful tool to evaluate novel therapies in a preclinical setting [80]. In a 3D setting, this technique can be used to study the effect of a given gene manipulation on an individual cell type within its cellular and ECM context, e.g. the contribution of a disease-promoting gene in epithelial compared to stroma or immune cells.

Mesenchymal stem cells (MSC). MSCs are multipotent stem cells found in various tissues, including bone marrow, placenta, adipose tissue, and endometrial tissue. Bone marrow is recognized as a source of endometrial regeneration, supported by its ability to produce decidual-like stroma during the activation of the protein kinase cAMP pathway *in vitro* [81].

Stem cell-driven 3D models could revolutionize the ability to study and fight endometriosis by building models that are a representative versions of endometrial tissue and lesions. For example, the 3D model created by Cheung et al. represents a hormonally responsive model with the use of human pluripotent stem cells together with endometrial stromal fibroblasts (PSC-ESFs) [82,83]*.* Stem cell studies are promising in developing markers targeted explicitly to them or applying stem cells to improve the specificity of the existing markers [81,84]. The multipotent potential and immunomodulatory activity of MSC make them ideal candidates for cell-based therapies. Endometrial stem cells with excellent proliferation, differentiation, and regenerative capacities, analogous to endometrial stem cells, have been identified in menstrual blood and effectively distinguished into endometrial cells using estrogen and progesterone therapy in *in vivo* rodent models. This supports the stem cell theory that describes how endometrial lesion formation occurs [33]. The use of stem cell in the 3D *in vitro* approach is vital in understanding the microenvironmental cues that trigger differentiation of endometriotic cells [15].

Current endometriosis research suffers from translational gaps, as the discovery of novel molecular mechanisms in traditional cell models and animal models needs to be translated into the development of novel therapeutic approaches [11,15]. Notably, as endometriosis is a heterogenous disease, such approaches have to consider patient outcomes, e.g. regarding fertility or pain scores. The incorporation of primary cells of patients with different manifestations of endometriosis (stratified by subtype, ASRM or #ENZIAN classification) and outcome into 3D *in vitro* models of endometriosis will allow for a systematic and patient subgroup-specific evaluation of molecular mechanisms and therapeutic targets.

The 3D *in vitro* models help to reduce the reliance on experimental animal models. This makes them a powerful tool for precision medicine. *Ciprietti* et al. studied gene expression on Asherman's syndrome (AS) using organoids derived from the endometrium of the patients with AS. In this example the organoids model played a key role in studying the gene expression changes in the WNT and NOTCH pathways. The *vitro* models provides a clinical translation where organoids derived from patients with endometriosis could be used to predict the treatment responses as it has been observed in the tumor organoids. Moreover, the model by *Boretto* et al. show that the disease specific organoids could be vital in capturing the clinical heterogeneity and understanding patient specific drug responses [21]. Therefore, the comparison of the derived organoids from healthy and diseased tissues will reveal the secreted factors including the genes expressed [78].

Recent studies on protein expression presented differences in the blood or urine of endometriosis patients compared to healthy controls or when compared to eutopic and ectopic endometrium samples [85]. The potential role of protein markers like vimentin has supported the hypothesis that cancer and endometriosis may share commonalities at the molecular level. Currently, there is increased observation of cancer-associated genes at specific chromosomal loci, along with their gene products [86]. Endometrial proteomic data with single-cell transcriptomic analyses and spatial transcriptomics are promising, especially in getting into the cellular heterogeneity and molecular mechanisms underlying endometriosis [87]. Indeed, there is an emphasis on the significance of proteomic approaches in discovering crucial biomarkers that could enhance diagnostic accuracy. 3D *in vitro* models of endometriosis provide a vast range of experimental settings for the functional evaluation of biomarkers, employing microRNA-, siRNA, or plasmid-based inhibition and overexpression approaches for selected biomarker candidates [15]. Such studies will complement the diagnostic function of biomarkers by a preclinical evaluation of their suitability as therapeutic targets.

The drive of any model system is to create a mimicry that serves as a more physical, theoretical, or mathematical representation of a real phenomenon that is difficult or impractical to detect directly. Computer simulations replicate a system's dynamic response by imitating the behavior of another system modeled after it. These simulations utilize mathematical descriptions and equations in the form of programs, generating a resulting data set upon running. By integrating bioengineering, computational modeling, and artificial intelligence (AI), researchers can develop a more realistic mimicry of the endometrium to aid in understanding potential therapeutic targets and factors contributing to endometriosis.

Computational models are gaining popularity for examining how various factors like immune, hormonal, and vascular disruptions may occur *in silico* and possibly lead to the development of the endometriosis condition. In this regard, the use of AI in endometriosis research focuses on three areas: predicting outcomes in endometriosis populations, creating diagnostic models, and enhancing research efforts [88]. AI has shown potential in disease diagnostics, advanced data analysis, and treatment. This has provided opportunities to decrease the time needed for the diagnosis and treatment of endometriosis [88,89].

Mbuguiro et al. highlight the use of regression, pharmacokinetics, and quantitative systems pharmacology modeling approaches that contribute to the research, diagnosis, and treatment of endometriosis [75]. The integration of AI tools with various 3D models has the potential to enhance our understanding of endometriosis through multi-omics approaches, leading to the generation of high-throughput data analysis that provides deeper insights into the complexities of the condition [88]. Mathematical models have been used in human cancer studies to quantify cancer morphology and progression through point-pattern analysis [90]. Building on the strengths of AI in pattern recognition, similar approaches could be applied for a subtype-specific analysis of endometriotic lesions and extraction of defining parameters that could aid their modeling in 3D models. Moreover, the integration of AI into 3D *in vitro* workflows will enhance reproducibility, scalability, and patient-specific customization.

## 5 Conclusion

Endometriosis is a complex disease that affects women of reproductive age. The progression of endometriosis involves several critical processes, including implantation, proliferation, invasion, angiogenesis, and apoptosis resistance. During disease progression, various signaling pathways contribute to the complexity of the condition. Advances in modeling endometriosis using *in vitro* 3D models offer a significant solution for better mimicking the human endometrium. This could potentially revolutionize our understanding of endometriosis pathophysiology and facilitate the identification of new targets for developing practical therapeutic approaches [14].

Primary cells and cell lines have been utilized in studies employing these models, as shown in Table 1. Some endometriosis models require a 3D matrix to provide a solid phase. Recently, various models have developed different 3D matrices for different cell types or cell lines to ensure solid phase support and strengths, as highlighted in Table 1. Notably, the purpose of the study and other factors determine the appropriate model to use. Spheroid scaffold-free model systems constructed from various cell lines have been predominantly used in studies of cell aggregation, invasion, and migration. For example, the St-T1b spheroids have demonstrated altered expression of genes involved in migration and invasion. The distinct invasion and migratory behavior of stromal cells have been observed in co-culture comparisons of stromal spheroids on Matrigel and Collagen I. For angiogenesis studies, CAM/AM, EN-MSCs, and HUVECs are preferred. However, progress has been made in integrating vascular systems into other 3D models. Technologies like bioprinting and microfluidics have advanced, enhancing efforts to integrate angiogenesis [14]. Additionally, the development of patient-derived organoids provides researchers with a robust system to further investigate gene expression patterns and signaling pathways associated with endometriosis, as well as their potential for future drug screening. 3D models reveal key physiological and molecular differences between healthy and diseased cells [44,63].

Compared to 2D endometrial models, 3D models have greatly transformed endometriosis research by offering more valuable investigative insights, despite each model presenting unique limitations (summary in Table 2). Nonetheless, 3D *in vitro* models collectively enhance our understanding of endometriosis even though none of the models fully replicate the complexities of the disease.

Moreover, there is a need for a multidisciplinary approach to create better models. Integrating computational modeling and bioengineering to develop AI-driven models that offer more informative and physiologically accurate mimics of the endometrium *in vivo* relies on identifying key molecular factors driving lesion formation, proliferation, and personalized medicine. AI can predict disease progression, patient outcomes, and drug responses, allowing for data-driven decision-making. Notably, in the coming years, the fusion of organoids and organ-on-a-chip technology and other 3D models explained above, gives hope that coupling bioengineering, AI gives hope for new insights into disease mechanisms and guidance to more effective and patient-tailored therapies.

In conclusion, we review the commonly used 3D models in endometriosis studies, highlighting their strengths and challenges and alluding to some of the future modifications to these models. These modifications focus on integrating multiple approaches to address the limitations discussed in this review, which will refine systems to enable researchers to model endometriosis more effectively under optimal physiological conditions *in vitro* to closely and accurately mimic the *in vivo* conditions.

## CRediT authorship contribution statement

**Cara Juli:** Investigation, Visualization, Writing – original draft. **Edward Mairura Nyang'au:** Funding acquisition, Investigation, Visualization, Writing – original draft. **Martin Götte:** Conceptualization, Funding acquisition, Supervision, Writing – review & editing. **Frauke von Versen-Höynck:** Conceptualization, Funding acquisition, Supervision, Writing – review & editing.

## Declaration of competing interest

The authors declare that they have no known competing financial interests or personal relationships that could have appeared to influence the work reported in this paper.

## Data Availability

No data was used for the research described in the article.
